# Root canal instrumentation of artificial primary teeth with rotary and reciprocating files: a micro-CT analysis

**DOI:** 10.1590/1807-3107bor-2025.vol39.116

**Published:** 2025-11-07

**Authors:** Daniela Alvim CHRISOSTOMO, Marcelle DANELON, Renan Diego FURLAN, Marco Antônio Hungaro DUARTE, Anna Carolina Volpi MELLO-MOURA, Cristiane DUQUE

**Affiliations:** (a) Universidade Estadual Paulista – Unesp, School of Dentistry, Department of Preventive and Restorative Dentistry, Araçatuba, SP, Brazil.; (b) Technische Universität Dresden, Medical Faculty Carl Gustav Carus, Policlinic of Operative Dentistry, Periodontology and Pediatric Dentistry, Dresden, Germany.; (c) Universidade de São Paulo – USP, School of Dentistry, Department of Operative Dentistry, Endodontics, and Dental Materials, Bauru, SP, Brazil.; (d) Universidade Católica Portuguesa – UCP, Faculty of Dental Medicine, Centre for Interdisciplinary Research in Health, Viseu, Portugal.

**Keywords:** Pulpectomy, Dental Instruments, Tooth, Deciduous

## Abstract

This study compared the outcomes of two endodontic instrumentation protocols (rotary or reciprocating files), using artificial primary teeth and micro-computed tomography. Twenty-four artificial primary molars were equally distributed into two groups of 12, according to the type of instrumentation – rotary files (Sequence Baby NiTi Files©) or reciprocating files (X1-Blue File NiTi files©). The following parameters were evaluated: root canal and dentin volumes, canal transportation and centering ability, risk of root perforation, and time of instrumentation. Statistically significant differences between the two instrumentation protocols considering root canal transportation and centering ability were identified. There was no difference in dentin thickness, fractures, and cracks comparing preoperative and postoperative time points for both endodontic files. Although the time of instrumentation was shorter for reciprocating files, rotary files promoted smaller root canal enlargement. Instrumentation with reciprocating and rotary files proved generally safe for pulpectomy in primary molars, promoting a negligible reduction in dentin volume, canal transportation, and centering ability, thereby preserving dentin thickness and lowering the risk of fractures.

## Introduction

New methods for root canal debridement and shaping have been studied to simplify and reduce the instrumentation time in primary teeth.^1^ Some *in vitro* and *in vivo* studies have evaluated the efficiency of rotary endodontic files compared to conventional files in pediatric patients.^2-5^ In *in vitro* studies, some parameters have been used to evaluate the efficacy of biomechanical preparation of primary or permanent teeth, such as shaping ability, root canal transportation, amount of dentin removal, untouched canal surface area, and preparation time.^3,4^ Most investigations have demonstrated better root canal shaping, lower dentin wear, and shorter working time using rotary files compared to hand files.^3,4^ The comparison between rotary and reciprocating files has shown that reciprocating instrumentation systems in permanent teeth lead to faster mechanical preparation of the root canal and a greater amount of dentin debris.^6^ The debris produced by reciprocating files are likely due to their cross-sectional design, higher cutting capacity, and greater dentin removal.^7^ In primary teeth, few *in vitro* studies have compared rotary and reciprocating files, with divergent results possibly stemming from methodological differences.^1,3,8^ Some studies have shown larger canal transportation and debris accumulation with reciprocating instrumentation,^3,8^ whereas other studies have not found reciprocating files to be more efficient considering canal transportation and average instrumentation time.^1^

Cone-beam computed tomography (CBCT) has been used in endodontics to generate three-dimensional images of teeth, helping clinicians to analyze root canal morphology more accurately and without the superimposition of anatomical structures.^9^ It is important to note, however, that CBCT is a clinical tool primarily used for diagnostic purposes. On the other hand, microcomputed tomography (micro-CT) produces ultrahigh-resolution 3D images with very thin sections (up to 1µm), which allows for a more detailed assessment of root canal anatomy. Micro-CT is mainly used in laboratory settings and is ideal for *in vitro* studies, offering the ability to perform precise comparisons between groups.^10,11^ These two techniques are distinct methodologies and not directly comparable, given their different applications and resolutions. An *in vitro* study demonstrated that micro-CT provided highly detailed and accurate data on root canal systems of primary teeth.^12^ On the other hand, micro-CT can only be applied in laboratory settings and it is unsuitable for clinical use because of its extremely high radiation levels, which are unsafe for humans.^9^

Studies comparing different root canal instrumentation systems in primary teeth are extremely limited due to the difficulty in collecting natural teeth with adequate root length, given the signs of root resorption typically observed when these teeth are prematurely extracted as a result of dental pathology, resulting in the exclusion of these specimens from the studies.^8^ To overcome this limitation, artificial primary teeth with coronal and root pulp have been used to evaluate the effectiveness of new endodontic rotary files in primary teeth.^5,8,13^

Several types of artificial teeth are available as root canal simulators such as three-dimensional printed replicas,^14,15^ resin teeth,^16^ ceramics, and plastic blocks.^17,18^ Artificial teeth are currently used for teaching various root canal treatment techniques for reproducing the features of natural teeth. In addition, artificial teeth do not offer a risk of infection, are available in large numbers, allow for a validated assessment through their uniformity, and offer different anatomical challenges, enabling the learning of standardized procedures.^16^

Considering the anatomical specificities of primary teeth and large variety of endodontic systems, including a new generation of heat-treated nickel-titanium (NiTi) files available in the market, new studies are essential for establishing root instrumentation protocols specifically designed for primary dentition. The files selected for this study are supported by the literature, but direct comparative studies between them are still lacking. The objective of this study was to compare the outcomes of two endodontic instrumentation protocols using rotary and reciprocating files, both with heat-treated NiTi technology, in artificial primary teeth and maxillary and mandibular primary molars, assessed by micro-CT. The null hypothesis is that there would not be differences between the two endodontic instrumentation protocols considering all evaluated parameters.

## Methods

### Teeth preparation and instrumentation methods

Sample size was calculated based on data from previous studies^5,12^using the freely available G*Power software (v3.1.9.2), resulting in at least 25 canals per group, and to account for possible losses, 30 canals were allocated to each group (n = 12 teeth per group). Twenty-four maxillary and mandibular artificial primary molars with coronal and root pulp (Denarte, São Paulo, Brazil) were selected and distributed into two groups of 12 (six maxillary and six mandibular primary molars), according to the type of instrumentation. Coronal access to the root canals was performed by a single operator with a high-speed water-cooled diamond bur (#FG1012, KG Sorensen Indústria e Comércio, São Paulo, SP, Brazil), followed by a conical steel bur with inactive tips (Endo Z, Maillefer Instruments, Ballaigues, Switzerland).^19^ The working length (WL) was determined with a size #10 K file placed 2.0 mm short of the apical foramen.^5^ The first group of artificial molars (n = 12) was instrumented with the SBF-Sequence Baby NiTi files© (SBF) (MKLife, Porto Alegre, Brazil) following the manufacturer’s recommended sequence: #17/08 (11 mm), #20/04, #25/04, and #30/04 (16 mm) with 350 rpm and 1.5 N torque. The second group of artificial teeth was initially instrumented with X1-Blue File Glide Path© (MKLife, Porto Alegre, Brazil) #15/04 (25 mm) and then with X1-Blue File© (MKLife, Porto Alegre, Brazil) reciprocating NiTi files (XBF) #25/06 (25 mm) with three pecking motions. All files were attached to an endodontic motor (E-connect Pro©, MKLife) and the teeth were irrigated with 3 mL of saline solution between each file change, using a 20G NaviTip needle (Ultradent, Indaiatuba, São Paulo, Brazil) inserted up to 2 mm with simultaneous aspiration. The canals were dried with sterile absorbent paper points. Instrumentation time was recorded in seconds using a chronometer.^4,13^

### Micro-CT registration

**Figure 1:**
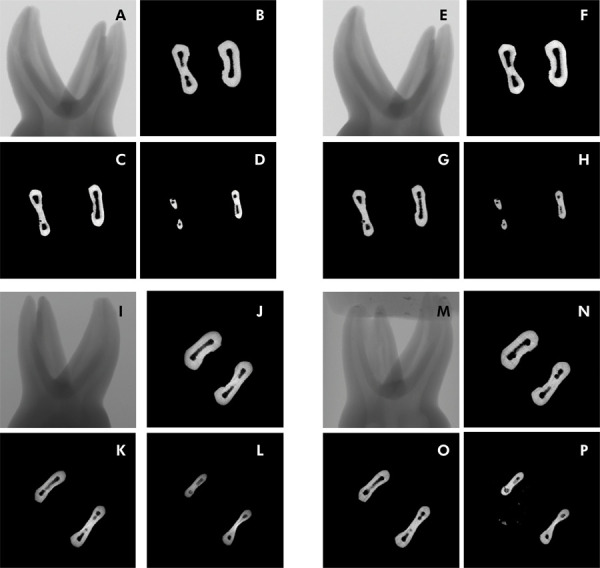
Representative micro-CT images of teeth before and after instrumentation with rotary and reciprocating files. A,E – Mandibular second molar before and after rotary instrumentation; B, F – cervical third of the same tooth before and after rotary instrumentation; C,G – middle third of the same tooth before and after rotary instrumentation; D,H - apical third of the same tooth before and after rotary instrumentation; I,M – Mandibular second molar before and after reciprocating instrumentation; J, N – cervical third of the same tooth before and after reciprocating instrumentation; K,O – middle third of the same tooth before and after reciprocating instrumentation; L,P - apical third of the same tooth before and after reciprocating instrumentation.

Micro-CT registration was conducted as described in previous studies^13,20 -24^ and performed by the same operator. Briefly, the teeth were scanned before and after mechanical preparation with a high-energy microcomputed tomograph (SkyScan 1272; Bruker Micro-CT, Kontich, Belgium) using the following acquisition parameters: 70 kV X-ray tube voltage, 142 µA anode current, and a voxel size of 14 μm. Only one specimen was scanned at a time. Scans were acquired at a resolution of 1,344 × 896 pixels, using a 0.5 mm-thick AI filter, 880 ms exposure, frame average of 3, random movement of 10. Images were captured with a 0.8° rotation step over 180°, resulting in a total scan time of approximately 23 min. A flat-field correction was performed before the scanning procedure to correct variations in camera pixel sensitivity. The images obtained before and after preparation were reconstructed using NRecon software (NRecon v.1.6.3; Bruker-microCT) and the following parameters: post-alignment, smoothing, ring artifacts, beam hardening corrections, and contrast limits set between 0.04 and 0.5. A data viewer software (Data Viewer v.1.5.1, Bruker, Kontich, Belgium) was used to pair and standardize the same position of specimens for all analyses. Quantitative analyses were then made by the same operator using CTAn software (CTAn v.1.14.4, Bruker, Kontich, Belgium) by means of mathematical operations, as described by Marciano et al.^24^ The total length of each canal was measured and split into three equal parts corresponding to the canal thirds (coronal, middle, and apical). Figure 1 shows micro-CT reconstructions and cross-sections at all levels of representative samples evaluated before and after instrumentation using rotary and reciprocating file protocols.

### Root canal and dentin volume measurements

In this study, although artificial primary molars are resin-based (with an undisclosed composition), the term “dentin” was used to facilitate comparison with previous studies. Root canal initial and final volume and dentin initial and final volume were obtained for each canal third before and after preparation. The percentage of root canal volumetric increase (% volumetric increase) and the percentage of root dentin volumetric decrease (% volumetric decrease) were calculated using the following formulas:^20,25^


$$\%\text{ root canal volumetric increase }=\left(\frac{\text{ Final volume of root canal }\times 100}{\text{ Initial root canal volume }}\right)-100$$

$$\%\text{ dentin volumetric decrease }=\left(\frac{\text{ Initial dentin volume }\times 100}{\text{ Final dentin volume }}\right)-100$$

### Measurement of root canal transportation (RCT) and centering ability (CA)

**Figure 2:**
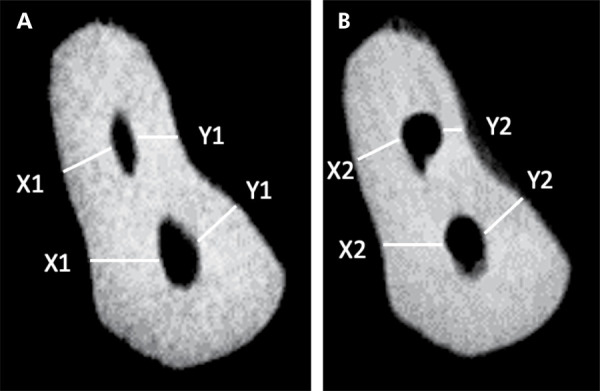
Representative micro-CT cross sections of the middle third of the root, taken from the mesial root canals of mandibular molar, showing the shortest distance between the edge of the root and the canal, used to assess canal transportation and centering ability. A. Preoperative root canal and B after root canal preparation.

These analyses were performed on the superimposed images, using the CTAn software. The RCT was calculated according to the following formula, proposed in a previous study:^26^ (X1-X2)-(Y1-Y2), where X1 and Y1 measurements represented the shortest distance from the external surface of the root to the periphery of the instrumented root canal before the preparation, for the mesial and distal surface, respectively; X2 and Y2 measurements represented the shortest distance from the external surface of the root to the periphery of the instrumented canal after the preparation, for the mesial and distal surface, respectively (Figure 2).^20,26^ A value of 0 indicated no canal transportation. A positive value indicated transportation toward the mesial root surface, corresponding to the side facing the furcation area (inner curve), while the negative value showed transportation toward the distal surface, opposite the furcation area (outer curve).^21^ CA was calculated using the following ratio, as proposed in a previous study:^26^ (X1-X2)/(Y1-Y2) or (Y1-Y2)/(X1-X2). The lower value was used as the numerator in the formula, when the two values were unequal, with 1 indicating perfect centering.^21,25^ Negative values represented deviation in the mesial direction, and positive numbers, in the distal direction.^20,25^

### Measurement of root perforation risk (dentin thickness, cracks, and perforations)

The smallest dentin thickness at the cervical, middle, and apical thirds after each instrumentation procedure was obtained using a 3D method. Dentin thickness between the root canal and the external surface was mapped and quantified in millimeters using the CTAn software (CTAn v.1.14.4, Bruker, Kontich, Belgium). The amount of dentin removal from each third was calculated by subtracting the thicker dentin thickness from the thinner dentin thickness before and after instrumentation.^21^ Using the CTAn software (CTAn v.1.14.4, Bruker, Kontich, Belgium), cracks and perforations were recorded by examining the 2D slices for each instrumented image stack ,comparing them to the baseline specimens.^8^

### Statistical analysis

The Shapiro-Wilk test was applied to verify the normality of the data. All data were subjected to ANOVA and Tukey’s tests at a 5% significance level. JAMOVI statistical software, version 2.3.19.0, was used for running the statistical analyses. The presence of cracks and perforations along the canal was recorded and qualitatively described.

## Results

### Root canal and dentin volume

As shown in Table 1, regardless of the type of molar or canal analyzed, a significant difference was observed between rotary (SBF) and reciprocating (XBF) files in terms of the increase in root canal volume. Rotary files promoted smaller root canal enlargement (p < 0.05), with reduced dentin removal compared with reciprocating files. The decrease in dentin volume was also higher for canals treated with XBF, but no difference was detected for distal canals when all molars were analyzed together. When maxillary and mandibular molars were analyzed separately, the same differences in root canal volume between the endodontic files were observed for all canals, except for the mesial canal of maxillary molars. Comparing the root canals, the highest increase in canal volume was observed for distal canals of maxillary molars and mesial canal of mandibular molars treated with XBF. For rotary files, the highest increase in canal volume was observed for both mesial and distal canals of maxillary molars, with no significant differences between them. No difference was noted between mesial and distal canals of mandibular molars treated with SBF. When the molars were analyzed separately, XBF promoted a significant decrease in dentin volume only for the distal canal of mandibular molars, compared to SBF.

**Table 1:** Increase in root canal volume (%) and decrease in dentin volume (%) after instrumentation with rotary (SBF) and reciprocating (XBF) files.

Teeth	Canals	Increase in canal volume (%)		Decrease in dentin volume (%)	
		SBF	XBF	SBF	XBF
Maxillary molars	Mesial	39.53(11.54)^Aa^	41.5 (12.9)^Aa^	5.24 (2.7)^Aa^	5.75 (2.62)^Aa^
	Distal	32.96(24.05)^Aa^	55.42(5.25)^Bb^	7.7(4.1)^Aa^	9.8 (7.26)^Aa^
	Palatal	4.23 (10)^Ba^	14.08 (10)^Cb^	3.19(1.3)^Aa^	5.04 (3.6)^Aa^
Mandibular molars	Mesial	14.65(5.29)^Aa^	35.79 (6.27)^Ab^	4.26(2.52)^Aa^	11.38 (8.81)^Aa^
	Distal	9.35(5.42)^Aa^	24.15 (2.97)^Bb^	3.36(0.89)^Aa^	5.41 (3.9)^Ab^
All molars	Mesial	27.1 (15.4)^Aa^	38.6(10.4)^Ab^	4.8(2.6)^Aa^	8.6(6.9)^Ab^
	Distal	21.2(20.9)^Aa^	39.8(29.2)^Ab^	5.5(3.7)^Aa^	7.6(6.1)^Aa^

SBF: Sequence Baby File NiTi©; XBF: X1-Blue File NiTi©; ^A^ Superscript uppercase letters in the same column indicate statistical difference among canals for each endodontic file system; ^a^Different superscript lowercase letters in the same row indicate statistical difference between endodontic files. Values are presented in means/standard deviations (ANOVA and Tukey’s tests, p <0.05).

### Root canal transportation (RCT) and centering ability (CA)

In general, neither of the instrumentation protocols presented statistical differences in root canal transportation and centering ability (Table 2). While both instrumentation systems yielded negative transportation values or , indicating deviation toward the opposite side of the furcation area; most values were close to zero. With respect to instrumentation, most results for both endodontic files showed canal deviation toward the opposite side of the furcation area, regardless of the protocol used. Statistical differences in canal transportation among the canal thirds were evidenced comparing both cervical and middle thirds with apical thirds, especially for specimens treated with rotary files. These differences can be seen for all canals of maxillary molars and distal canals of mandibular molars. During the shaping procedure, no files were broken or deformed.

**Table 2:** Root canal transportation and centering ability after instrumentation with rotary (SBF) and reciprocating (XBF) files.

Teeth	Canals	Thirds	Root canal transportation^*^		Centering ability**	
			SBF	XBF	SBF	XBF
Maxillary molars	Mesial	Cervical	-0.15^Aa^	-0.14^Aa^	0.09 ^Aa^	0.2 ^Aa^
			(-0.23; -0.05)	(-0.96; -0.02)	(-0.6;0.26)	(-0.019;0.65)
		Middle	-0.02^ABa^	-0.002^Aa^	0.47 ^Aa^	0.33 ^Aa^
			(-0.24;0.09)	(-1.03;0.16)	(-0.97;0.9)	(-2.33;0.96)
		Apical	-0.008^Ba^	-0.039^Aa^	0.33 ^Aa^	-0.06 ^Aa^
			(-0.07;0.10)	(-0.59;0.18)	(-0.23;0.8)	(-5.2;0.87)
	Distal	Cervical	-0.14^Aa^	-0.2^Ab^	0.2^Aa^	0.053^Ab^
			(-0.19;-0.08)	(-0.3;-0.15)	(-0.1;0.5)	(-0.21;0.23)
		Middle	-0.004^ABa^	-0.02^ABa^	0.49 ^Aa^	0.25 ^Aa^
			(-0.21;0.09)	(-0.3;0.04)	(-0.7;1)	(-1.23;2.74)
		Apical	0.06^Ba^	-0.01 ^Ba^	0.27 ^Aa^	0.2 ^Aa^
			(-0.07;0.18)	(-0.10;0.58)	(-0.4;0.84)	(-1.04;0.97)
	Palatal	Cervical	-0.14^Aa^	-0.15 ^Aa^	0.19 ^Aa^	-0.33 ^Aa^
			(-0.23;0.01)	(-0.31;0.08)	(-0.9;0.62)	(-0.83;0.04)
		Middle	-0.064^ABa^	-0.03 ^Aa^	-0.12 ^Aa^	-0.23 ^Aa^
			(-0.09;011)	(-0.38;0.25)	(-1.72;0.5)	(-2.79;1.88)
		Apical	0.13^Ba^	0.01 ^Aa^	-0.04 ^Aa^	-0.06 ^Aa^
			(-0.04;0.88)	(-0.97;0.10)	(-0.3;0.01)	(-0.6;1.5)
Mandibular molars	Mesial	Cervical	-0.07 ^Aa^	-0.04 ^Aa^	-0.21 ^Aa^	0.11 ^Aa^
			(-0.19;0.05)	(-0.17;0.08)	(-2.23;1.8)	(-1.14;1.24)
		Middle	0.026 ^Aa^	-0.05 ^Aa^	0.08 ^Aa^	-0.21 ^Aa^
			(-0.11;0.19)	(-0.17;0.11)	(-1.4;1.97)	(-3.07;2.11)
		Apical	-0.007 ^Aa^	-0.01 ^Aa^	0.32 ^Aa^	0.29 ^Aa^
			(-0.07;0.09)	(-0.08;0.07)	(-1.5;2.42)	(-0.19;1.4)
	Distal	Cervical	-0.12^Aa^	-0.046 ^Aa^	-0.17 ^Aa^	0.069 ^Aa^
			(-0.23;0.84)	(-0.39;0.16)	(-3.1;2)	(-2.1;0.90)
		Middle	-0.03^ABa^	-0.11^Ab^	-0.48 ^Aa^	-0.38 ^Aa^
			(-0.12;0.10)	(-0.18;0.01)	(-4.2;0.3)	(-2.32;0.66)
		Apical	0.05^Ba^	-0.4 ^Aa^	-0.2 ^Aa^	0.14 ^Aa^
			(-0.10;0.18)	(-0.06;0.13)	(-1.5;2.3)	(-1.76;1)
All molars	Mesial	Cervical	-0.09^Aa^	-0.06 ^Aa^	0.021^Aa^	0.21^Ab^
			(-0.23;0.05)	(-0.96;0.08)	(-2.2;1.8)	(-1.14;1.24)
		Middle	-0.011^ABa^	-0.037 ^Aa^	0.11 ^Aa^	0.13 ^Aa^
			(-0.24;0.19)	(-1.03;0.16)	(-1.4;1.9)	(-3.07;2.11)
		Apical	-0.008^Ba^	-0.023 ^Aa^	0.33 ^Aa^	0.16 ^Aa^
			(-0.07;0.10)	(-0.59;0.18)	(-1.5;2.4)	(-3.4;1.4)
	Distal	Cervical	-0.14 ^Aa^	-0.18 ^Aa^	0.05 ^Aa^	0.06 ^Aa^
			(-0.23;0.84)	(-0.39;0.16)	(-0.7;0.5)	(-2.1;0.9)
		Middle	-0.008 ^Aa^	-0.06 ^ABb^	-0.035 ^Aa^	0.07 ^Aa^
			(-0.21;0.10)	(-0.31;0.04)	(-4.28;1)	(-2.3;2.74)
		Apical	0.05 ^Ba^	-0.03^Ba^	-0.12 ^Aa^	0.21 ^Aa^
			(-0.10;0.18)	(-0.10;0.58)	(-1.5;2.3)	(-1.76;1)

SBF: Sequence Baby File NiTi©; XBF: X1-Blue File NiTi©; ^A^Superscript uppercase letters in the same column indicate statistical difference among the thirds for each canal and endodontic file system, separately. ^a^Different superscript lowercase letters in the same row indicate statistical difference between endodontic files. Values are presented in medians (minimum; maximum) (Kruskal-Wallis test and Dunn’s test, p < 0.05). ^*^Regarding root canal transportation, negative values indicate transportation to the opposite side of the furcation area; positive values indicate transportation towards the furcation area. Zero indicates that no canal transportation occurred. **Regarding centering ability, negative values represent deviation towards the furcation area, while positive values indicate deviation in the opposite direction. A value of 1 indicates perfect centering ability.^21^

### Risk of root perforation (dentin thickness, cracks, and perforations)

When all molar types were assessed together, no difference in dentin thickness was found between preoperative and postoperative time points for either endodontic file, except at the cervical third of the mesial canals (Table 3). Statistical differences among the thirds were observed for all canals treated at both time points, independently of the group, except for mesial canals treated with SBF. For maxillary molars, statistical differences between preoperative and postoperative time points were observed for all thirds of mesial canals and cervical thirds of distal and palatal canals treated only with SBF. There were statistical differences among the thirds for most of canals treated at preoperative and postoperative time points for both files. For mandibular molars, statistical differences between preoperative and postoperative time points were not observed for any group. Statistical differences among the canals thirds were observed in all mandibular molars at both time points, regardless of the group, except for mesial canals instrumented with rotary files (Table 3).

**Table 3:** Dentin thickness (mm) on inner root canals before and after instrumentation with rotary (SBF) and reciprocating (XBF) files.

Teeth	Canals	Thirds	SBF		XBF	
			Preoperative	Postoperative	Preoperative	Postoperative
Maxillary molars	Mesial	Cervical	0.55^Aa^	0.39^Ab^	0.60 ^Aa^	0.44 ^Ab^
			(0.08)	(0.10)	(0.11)	(0.15)
		Middle	0.44^Aa^	0.36^Ab^	0.43 ^Ba^	0.40 ^Aa^
			(0.13)	(0.08)	(0.12)	(0.15)
		Apical	0.33^Ba^	0.34^Ab^	0.32^Ca^	0.32 ^Aa^
			(0.10)	(0.08)	(0.11)	(0.11)
	Distal	Cervical	0.56^Aa^	0.36^Ab^	0.72 ^Aa^	0.47 ^Ab^
			(0.12)	(0.13)	(0.08)	(0.10)
		Middle	0.31^Ba^	0.23^Ba^	0.47 ^Ba^	0.44 ^Aa^
			(0.15)	(0.13)	(0.08)	(0.07)
		Apical	0.26^Ba^	0.24^Ba^	0.26 ^Ca^	0.24 ^Ba^
			(0.11)	(0.09)	(0.04)	(0.05)
	Palatal	Cervical	0.62^Aa^	0.49^Ab^	0.86 ^Aa^	0.75 ^Ab^
			(0.06)	(0.08)	(0.14)	(0.05)
		Middle	0.34^Ba^	0.35^Ba^	0.56 ^Ba^	0.55 ^Ba^
			(0.25)	(0.21)	(0.19)	(0.11)
		Apical	0.44^Ba^	0.47^Aa^	0.40 ^Ca^	0.44 ^Ca^
			(0.28)	(0.24)	(0.22)	(0.29)
Mandibular molars	Mesial	Cervical	0.59^Aa^	0.55^Aa^	0.74 ^Aa^	0.72 ^Aa^
			(0.17)	(0.16)	(0.18)	(0.15)
		Middle	0.41^Aa^	0.44^Aa^	0.52 ^A,Ba^ (0.14)	0.51 ^Ba^
			(0.13)	(0.14)		(0.10)
		Apical	0.43^Aa^	0.42^Aa^	0.45 ^Ba^	0.47 ^Ba^
			(0.14)	(0.14)	(0.11)	(0.07)
	Distal	Cervical	0.73^Aa^	0.67^Aa^	0.84 ^Aa^	0.77 ^Aa^
			(0.08)	(0.19)	(0.19)	(0.19)
		Middle	0.43^Ba^	0.41^Ba^	0.53 ^Ba^	0.44 ^Ba^
			(0.09)	(0.14)	(0.15)	(0.14)
		Apical	0.27^Ca^	0.32^Ca^	0.39 ^Ca^	0.35 ^Ba^
			(0.07)	(0.06)	(0.06)	(0.08)
All molars	Mesial	Cervical	0.55 ^Aa^	0.39 ^Aa^	0.60 ^Aa^	0.44 ^Ab^
			(0.08)	(0.10)	(0.11)	(0.15)
		Middle	0.44 ^Aa^	0.36 ^Aa^	0.43^Ba^	0.40 ^A,Ba^
			(0.13)	(0.08)	(0.12)	(0.15)
		Apical	0.44 ^Aa^	0.44 ^Aa^	0.32 ^Ca^	0.31 ^Ba^
			(0.10)	(0.10)	(0.11)	(0.11)
	Distal	Cervical	0.56 ^Aa^	0.36 ^Aa^	0.72 ^Aa^	0.47 ^Aa^
			(0.12)	(0.13)	(0.08)	(0.10)
		Middle	0.31^A,B,a^	0.23 ^Aa^	0.47 ^Ba^	0.44 ^Aa^
			(0.15)	(0.13)	(0.08)	(0.07)
		Apical	0.26 ^Ba^	0.24 ^Ba^	0.26 ^Ca^	0.24 ^Ba^
			(0.11)	(0.09)	(0.04)	(0.05)

SBF: Sequence Baby File NiTi©; XBF: X1-Blue File NiTi©; ^A^Superscript uppercase letters in the same column indicate statistical difference among the thirds for each canal and endodontic files, separately. ^a^Different superscript lowercase letters in the same row indicate statistical difference between preoperative and postoperative time points. Values are presented in means (standard deviations) (ANOVA and Tukey’s tests, p < 0.05).

**Figure 3:**
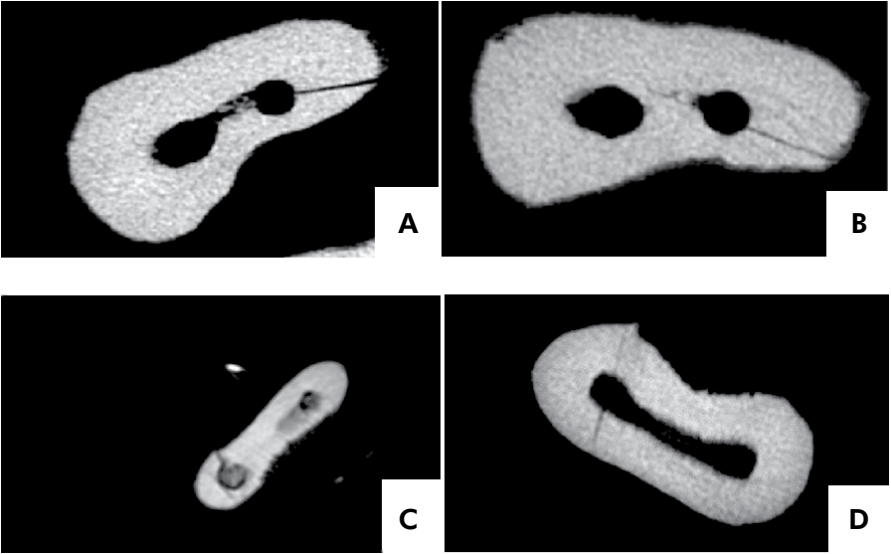
Representative micro-CT images of fractures and cracks detected after instrumentation with rotary and reciprocating systems.

A statistically significant difference in instrumentation time (p < 0.05) was found between the groups, with reciprocating files requiring the shortest time (Table 4). No statistical difference in the total number of fractures and cracks was found between the groups (p > 0.05). Root fractures were only observed in three specimens treated with SBF (five fractures) and in three specimens treated with XBF (five fractures), with more than one type of fracture in some of the specimens. Cracks were observed in only one specimen treated with XBF (Table 4). Fractures and cracks detected after instrumentation with rotary and reciprocating files are illustrated in Figure 3.

**Table 4:** Instrumentation time, fractures, and cracks detected after instrumentation with rotary (SBF) and reciprocating (XBF) files.

Variables	SBF	XBF
Instrumentation time (s)	399.25 (38.12) *	232.5 (23.07)
Total number of fractures and cracks (n of fractures/n of cracks)	Mesial – 3C, 1M, 1A/0	Mesial – 1C, 2M, 1A /0
Cervical (C), middle (M), and apical (A)	Distal - 0/0	Distal – 1C/1A
	Palatal - 0/0	Palatal – 0/0

SBF: Sequence Baby File NiTi^©^; XBF: X1-Blue File NiTi. *Statistical difference between endodontic files according to Student’s t test (p < 0.05).

A. Cervical third fracture after rotary instrumentation; B. Middle third fracture after rotary instrumentation; C. Apical third fracture after reciprocating instrumentation and D. Cervical crack after reciprocating instrumentation.

## Discussion

In the present study, the null hypothesis was not accepted because some differences were detected between rotary and reciprocating files in terms of the parameters analyzed on micro-CT images before and after instrumentation.

*In vitro* studies have been conducted to evaluate new root instrumentation techniques for primary teeth, assessing parameters such as efficacy, shaping ability, and canal transportation with rotary and reciprocating files using micro-CT analyses.^3,4,8,12^ Few *in vitro* studies have evaluated specific rotary files for primary teeth - Sequence Baby NiTi files© (SBF), reporting significantly larger dentin wear and shorter working time than manual instrumentation.^5, 13^

Considering the difficulty in collecting natural teeth with adequate root length,^8^ artificial primary teeth with coronal and root pulp have been used in some recent studies to test contemporary instrumentation systems, their application, and their effectiveness on primary teeth.^5,8,13^ In the present study, artificial teeth were chosen because of the advantages of having roots and canals with standardized length and width, reducing the variability associated with natural primary teeth that could interfere in the comparison between endodontic files.

Regardless of the type of molar and canal analyzed in the present study, instrumentation with rotary files (SBF-Sequence Baby NiTi files©) promoted smaller root canal enlargement and reduced dentin removal less when compared with reciprocating files (X1-Blue File reciprocating NiTi files©). The same was observed in the study by Morales et al.^11^ This aspect is important when evaluating the conservative performance of files; however, previous findings should also be considered, given that rotary files have been shown to contact only 50% of the canal walls.^27^ Conservative performance should not compromise the file’s ability to clean and remove pulp tissue remnants from the root canal system.^11^ In contrast to our findings, a previous study found no difference in volume increase between root canals prepared with rotary or reciprocating files.^28^ No guidelines suggesting an allowable amount of dentin removal in root canal preparation have been established, but excessive dentin removal can lead to failure and weakening of the remaining tooth structure.^4^ In the present study, the highest volume of dentin removed by reciprocating files (XBF) may be a result of their convex triangular cross-section and larger taper (#25/06) design. Comparing the root canals, the highest increase in canal volume was observed for distal or mesial canals compared to palatal canals of maxillary molars, regardless of the endodontic file used. Studies have shown that the amount of dentin removal can be influenced by file taper and size and by the root canal morphology of primary teeth.^2,29^ In the present study, dentin removal was likely less effective in palatal canals because of their larger volume compared to other canals of maxillary molars.

Canal transportation indicates excessive dentin removal from the canals and the formation of gaps/perforations; therefore, the selection of the ideal file can minimize this effect.^4,30^ NiTi rotary files, such as those used in our study, have become popular owing to their superior flexibility^31^ and centering ability^25^compared with stainless steel files. These features allow the files to follow the original anatomy of the curved canals of primary teeth, thus minimizing the risk of procedural errors.^32^ In particular, heat treatment changes the phase transformation temperature of the NiTi alloy, promoting the formation of soft and ductile phases, i.e., the martensite phase and R-phase, thereby enhancing fracture strength and flexibility.^33,34^ In the present study, no statistical differences were observed between the endodontic files regarding canal transportation and centering ability (Table 2), which is likely correlated with the greater flexibility of the files. Similar findings between rotary and reciprocating files were also observed by de Camargo et al.,^21^ and different results between those instruments were observed by Nazari Moghadam et al.^35^ In the present study, statistical differences in canal transportation among the canal thirds were evidenced comparing both cervical and middle thirds with apical thirds, especially for specimens treated with rotary files, unlike the results obtained by Nazari Moghadam et al.^35^ These findings can be explained by the fact that rotary files – Sequence Baby NiTi Files© - involves a sequence of four files with different tapers #17/08 (11 mm), #20/04, #25/04, and #30/04 (16 mm) for instrumentation. One study compared the cleaning and shaping effectiveness of primary root canals after instrumentation with rotary, sonic, and conventional instruments and found greater deviations at cervical third, followed by middle and apical thirds.^36^ In our study, both files promoted higher canal transportation towards the opposite side of the furcation area , with most values remaining close to zero, in line with those of a previous study.^35^ Moreover, canal transportation equal to or less than 0.1 mm is clinically acceptable.^30^ In summary, both endodontic files used during root canal preparation showed overall safety, because neither group excessively removed dentin from the cervical, middle, or apical thirds.

No difference in dentin thickness on the inner canal walls was observed between preoperative and postoperative time points for either endodontic file, however, differences among the thirds were observed for most of canals treated at both time points, with the apical third exhibiting the lowest values, as expected.

This can be explained by the anatomical characteristic of the canal, which increases the contact between the file and the conical surface of the root canal during insertion and instrumentation.^11^ A previous study has shown that maintaining a minimum dentin thickness of 0.3 mm in root canals instrumented with stainless steel files ensures overall safety and minimizes the risks of cracks and perforations.^37^ In the present study, root fracture was only observed in three specimens treated with SBF and in three specimens treated with XBF, with more than one type of fracture in some specimens. Notably, the teeth used in the present study and in a previous study^8^ did not share the same mechanical properties as those of the dentin of primary teeth. The difference in elastic modulus between the resin prototype material/artificial teeth (2GPa)^3^and primary root dentin (11.6GPa)^38^ indicates that the artificial teeth are likely more prone to elastic deformation than natural primary root dentin. Fractures and cracks may develop more rapidly in the dentin of primary roots than in artificial teeth.^8^

In this study, instrumentation time was shorter for reciprocating files compared to rotary files, as observed in previous studies.^4,12^ We used only two files (one for glide path and another one for root canal preparation) in the reciprocating protocol, unlike the rotary protocol, which required four files and more time for instrument change. Although there is no gold standard for endodontic files in pediatric dentistry, hand files are commonly used as a control group to assess the efficacy of rotary instrumentation. In the present study, we chose to compare two rotary instrumentation protocols, considering that most investigations and systematic reviews have reported better root canal shaping, shorter working time, and optimal rates of canal filling of primary teeth treated with rotary files compared to hand files.^3,4,12,13,32,39-41^

The limitations of this study may be attributed to potential minor defects in the manufacturing process of artificial primary teeth, affecting both the internal and external surfaces of the root canals. Moreover, the difference in hardness between human dentin and resin represents a limitation in the use of artificial teeth. Despite significant advances in the field, no material has yet been able to fully simulate human dentin.^42^ To minimize the impact of those limitations on the results, we used the same molars to evaluate preoperative and postoperative time points and the same type of maxillary and mandibular molars to compare the endodontic files. Another limitation is the difference between file size and taper in each instrumentation system; however, we focused more on “cleaning” than on “shaping” to minimize excessive dentin removal and used the appropriate movement amplitude within the root canal.^8^ Sample size could also be a limitation of the study, considering the calculation did not account for subgroup division (maxillary and mandibular molars); however, this has probably low impact on the general results, given the similarity of the artificial teeth in each group.

From a clinical perspective, this *in vitro* model is a valuable tool for evaluating different instrumentation techniques and systems before their recommendation for pulpectomies in human primary teeth, taking into account essential factors for clinical application, such as safety, efficiency, and preservation of the tooth structure. A notable finding of this study is the shorter instrumentation time achieved with the reciprocating system compared to the rotary system, a result that is highly relevant to pediatric dentistry. Faster procedures are crucial in pediatric dentistry due to young patients’ shorter attention spans and increased susceptibility to discomfort. The ability to complete the instrumentation process more quickly could significantly improve patient cooperation, reduce treatment time, and minimize the risk of anxiety and fatigue, contributing to a more positive experience during dental visits.

## Conclusion

Both rotary and reciprocating endodontic instrumentation protocols demonstrated overall safety for use in pulpectomy of primary teeth. Given the limited number of studies on endodontic treatment of primary teeth, the present study provides a foundation for future clinical studies.

## Data Availability

The authors declare that all data generated or analyzed during this study are included in this published article.

## References

[1] Prabhakar AR, Yavagal C, Dixit K, Naik SV (2016). Reciprocating vs rotary instrumentation in pediatric endodontics: cone Beam Computed Tomographic analysis of deciduous root canals using two single-file systems. Int J Clin Pediatr Dent.

[2] Kaya E, Elbay M, Yigit D (2017). Evaluation of the Self-Adjusting File system (SAF) for the instrumentation of primary molar root canals: a micro-computed tomographic study. Eur J Paediatr Dent.

[3] Moraes RD, Santos TM, Marceliano-Alves MF, Pintor AV, Lopes RT, Primo LG (2019). Reciprocating instrumentation in a maxillary primary central incisor: A protocol tested in a 3D printed prototype. Int J Paediatr Dent.

[4] Manker A, Solanki M, Tripathi A, Jain ML (2020). Biomechanical preparation in primary molars using manual and three NiTi instruments: a cone-beam-computed tomographic in vitro study. Eur Arch Paediatr Dent.

[5] Souza BK, Alcalde MP, Duarte MA, Machado MA, Oliveira TM, Lourenço N (2023). Shaping ability of a pediatric motor-driven instrumentation system in primary molar root canal prototypes. Braz Dent J.

[6] Uslu G, Özyürek T, Yilmaz K, Gündogar M, Plotino G (2018). Apically extruded debris during root canal instrumentation with Reciproc Blue, HyFlex EDM, and XP-endo Shaper Nickel-titanium Files. J Endod.

[7] Eshagh Saberi A, Ebrahimipour S, Saberi M (2020). Apical debris extrusion with conventional rotary and reciprocating instruments. Iran Endod J.

[8] Moraes RD, Perez R, Silva AS, Machado AS, Lopes RT, Pintor AV (2021). Micro-CT evaluation of root canal preparation with rotary instrumentation on prototyped primary incisors. Braz Oral Res.

[9] Acar B, Kamburoglu K, Tatar I, Arikan V, Çelik HH, Yüksel S (2015). Comparison of micro-computerized tomography and cone-beam computerized tomography in the detection of accessory canals in primary molars. Imaging Sci Dent.

[10] Peters OA, Schönenberger K, Laib A (2001). Effects of four Ni-Ti preparation techniques on root canal geometry assessed by micro computed tomography. Int Endod J.

[11] Morales ML, Sánchez JA, Elmsmari F, Duran-Sindreu F, Salmon P, Jaramillo DE (2024). Microcomputed tomographic evaluation of 6 NiTi files on the pericervical dentin and the smallest dentin thickness zones in mesial root canals of mandibular molars: an in vitro study. Clin Oral Investig.

[12] Barasuol JC, Alcalde MP, Bortoluzzi EA, Duarte MA, Cardoso M, Bolan M (2021). Shaping ability of hand, rotary and reciprocating files in primary teeth: a micro-CT study in vitro. Eur Arch Paediatr Dent.

[13] Rêgo EF, Silva RP, de Sá Silva AS, Marceliano-Alves MF, Lopes RT, Primo LG (2024). Instrumentation time and effectiveness of hand and rotary files in a prototyped second mandibular primary molar: a micro-CT study. Int J Paediatr Dent.

[14] Wolgin M, Wiedemann P, Frank W, Wrbas KT, Kielbassa AM (2015). Development and evaluation of an endodontic simulation model for dental students. J Dent Educ.

[15] Robberecht L, Chai F, Dehurtevent M, Marchandise P, Bécavin T, Hornez JC (2017). A novel anatomical ceramic root canal simulator for endodontic training. Eur J Dent Educ.

[16] Al-Sudani DI, Basudan SO (2017). Students' perceptions of pre-clinical endodontic training with artificial teeth compared to extracted human teeth. Eur J Dent Educ.

[17] Reymus M, Fotiadou C, Kessler A, Heck K, Hickel R, Diegritz C (2019). 3D printed replicas for endodontic education. Int Endod J.

[18] Tchorz JP, Brandl M, Ganter PA, Karygianni L, Polydorou O, Vach K (2015). Pre-clinical endodontic training with artificial instead of extracted human teeth: does the type of exercise have an influence on clinical endodontic outcomes?. Int Endod J.

[19] Pinto DN, Sousa DL, Araújo RB, Moreira-Neto JJ (2011). Eighteen-month clinical and radiographic evaluation of two root canal-filling materials in primary teeth with pulp necrosis secondary to trauma. Dent Traumatol.

[20] Pinto JC, Pivoto-João MM, Espir CG, Ramos ML, Guerreiro-Tanomaru JM, Tanomaru-Filho M (2019). Micro-CT evaluation of apical enlargement of molar root canals using rotary or reciprocating heat-treated NiTi instruments. J Appl Oral Sci.

[21] Camargo EJ, Duarte MA, Alcalde MP, Só MV, Vasconcelos BC, Silva EJ (2021). Safety of large preparation with different instruments in the buccal canals of maxillary molars. Aust Endod J.

[22] Hristov K, Gigova R, Gateva N, Angelova L (2024). Micro-computed tomography (micro-CT) evaluation of root canal morphology in immature maxillary third molars. J Clin Pediatr Dent.

[23] AbuMostafa A, Alrefaie MM, Abu-Mostafa N, Algahtani FN (2024). Microcomputed tomography analysis of curved root canal preparation when coronal flaring and glide path files used with heat-treated nickel titanium rotary files. PLoS One.

[24] Marciano MA, Duarte MA, Ordinola-Zapata R, Del Carpio Perochena A, Cavenago BC, Méndes-Vilas editor (2012). Current microscopy contributions to advances in science and technology.

[25] Neves AA, Silva EJ, Roter JM, Belladona FG, Alves HD, Lopes RT (2015). Exploiting the potential of free software to evaluate root canal biomechanical preparation outcomes through micro-CT images. Int Endod J.

[26] Gambill JM, Alder M, Rio CE (1996). Comparison of nickel-titanium and stainless steel hand-file instrumentation using computed tomography. J Endod.

[27] Pérez Morales ML, González Sánchez JA, Olivieri JG, Elmsmari F, Salmon P, Jaramillo DE (2021). Micro-computed tomographic assessment and comparative study of the shaping ability of 6 nickel-titanium files: an in vitro study. J Endod.

[28] Tavares SJ, Sarmento EB, Guimarães LD, Antunes LA, Antunes LS, Gomes CC (2019). The influence of kinematics of engine-driven nickel-titanium instruments on root canal shape assessed by micro-computed tomography: a systematic review. Acta Odontol Scand.

[29] Radhika E, Reddy ER, Rani ST, Kumar LV, Manjula M, Mohan TA (2017). Cone Beam Computed Tomography evaluation of hand nickel-titanium K-files and rotary system in primary teeth. Pediatr Dent.

[30] Peters OA (2004). Current challenges and concepts in the preparation of root canal systems: a review. J Endod.

[31] Ebihara A, Yahata Y, Miyara K, Nakano K, Hayashi Y, Suda H (2011). Heat treatment of nickel-titanium rotary endodontic instruments: effects on bending properties and shaping abilities. Int Endod J.

[32] Guelzow A, Stamm O, Martus P, Kielbassa AM (2005). Comparative study of six rotary nickel-titanium systems and hand instrumentation for root canal preparation. Int Endod J.

[33] Zupanc J, Vahdat-Pajouh N, Schäfer E (2018). New thermomechanically treated NiTi alloys - a review. Int Endod J.

[34] Agrawal PR, Chandak M, Nikhade PP, Patel AS, Bhopatkar JK (2024). Revolutionizing endodontics: advancements in nickel-titanium instrument surfaces. J Conserv Dent Endod.

[35] Nazari Moghadam K, Teimoori S, Labbaf H, Kazemi A, Kavosi A, Mahdavi F (2019). Canal transportation and centering after using Path File and R-Pilot in mesiobuccal canals of maxillary molars using cone-beam computed tomography. Iran Endod J.

[36] Shaikh SM, Goswami M (2018). Evaluation of the effect of different root canal preparation techniques in primary teeth using CBCT. J Clin Pediatr Dent.

[37] Lim SS, Stock CJ (1987). The risk of perforation in the curved canal: anticurvature filing compared with the stepback technique. Int Endod J.

[38] Angker L, Swain MV, Kilpatrick N (2003). Micro-mechanical characterisation of the properties of primary tooth dentine. J Dent.

[39] Faghihian R, Amini K, Tahririan D (2022). Rotary versus manual instrumentation for root canal preparation in primary teeth: a systematic review and meta-analysis of clinical trials. Contemp Clin Dent.

[40] Shroff Y, Dutta BN, Verma RK, Sharma V (2024). Comparative evaluation of the efficacy of the Pro AF Baby Gold and Kedo-S pediatric endodontic files for canal instrumentation, transportation, and centering ratio: a systematic review and meta-analysis. J Indian Soc Pedod Prev Dent.

[41] Padmawar N, Pawar N, Tripathi V, Banerjee S, Tyagi G, Joshi SR (2025). Comparative analysis of rotary versus manual instrumentation in paediatric pulpectomy procedures: A systematic review and meta-analysis. Aust Endod J.

[42] Reymus M, Stawarczyk B, Winkler A, Ludwig J, Kess S, Krastl G (2020). A critical evaluation of the material properties and clinical suitability of in-house printed and commercial tooth replicas for endodontic training. Int Endod J.

